# Multimodal imaging features of primary pericardial synovial sarcoma: a case report

**DOI:** 10.3389/fonc.2023.1181778

**Published:** 2023-08-04

**Authors:** Hongrui Jin, Yong Zhang, Wenbo Zhang, Keyan Wang

**Affiliations:** Department of Magnetic Resonance Imaging, The First Affiliated Hospital of Zhengzhou University, Zhengzhou, China

**Keywords:** pericardial synovial sarcoma, multimodal imaging, echocardiogram, computed tomography, cardiac magnetic resonance imaging

## Abstract

**Background:**

Primary pericardial synovial sarcoma is an extremely rare malignant tumor, and affected patients have a poor prognosis. Only a few cases have been reported in the literature.

**Case summary:**

A 34-year-old man was admitted to our hospital with chest tightness and a cough. An echocardiogram revealed a heterogeneous mass with a large pericardial effusion. Further computed tomography (CT) of the chest and cardiac magnetic resonance imaging (CMRI) demonstrated an irregular pericardial mass abutting the left atrium and left ventricle and invading the mediastinal structures. Pathology results showed that the tumor was a monophasic synovial sarcoma. The patient underwent chemotherapy and survived for 17 months.

**Discussion:**

Many cardiac tumors are clinically asymptomatic or nonspecific, and they are frequently detected or diagnosed at an advanced stage of the disease. Multimodal cardiac imaging facilitates the detection and assessment of cardiac tumors. In particular, CMRI is considered as a superior imaging tool, because it provides high tissue contrast and can detect invasion of the myocardium. We describe the clinical details and multimodal imaging features of a rare primary pericardial synovial sarcoma, hoping to provide guidance for the diagnosis of similar cases in the future.

## Introduction

Synovial sarcoma (SS) is a kind of aggressive, soft-tissue, and malignant sarcoma. The incidence of SS in soft tissues is about 10%, and it usually arises in the deep soft tissue of children and young adults, especially around limb joints and tendon tissues (1). Primary cardiac SS (CSS) is extremely rare, representing less than 5% of all primary cardiac sarcomas (2). Primary pericardial SS (PSS) is an even more rare malignant neoplasm. The presence of primary PSS portends a very poor prognosis, but surgical resection in combination with adjuvant chemotherapy and radiation has been found to significantly prolong survival (3). Multimodal imaging plays a fundamental role in detecting and assessing cardiac tumors, and it can be used to design individualized regimens and to assess prognosis. In this article, we describe the clinical manifestations and imaging features of a rare case of primary PSS.

## Case presentation

**Figure 1:**
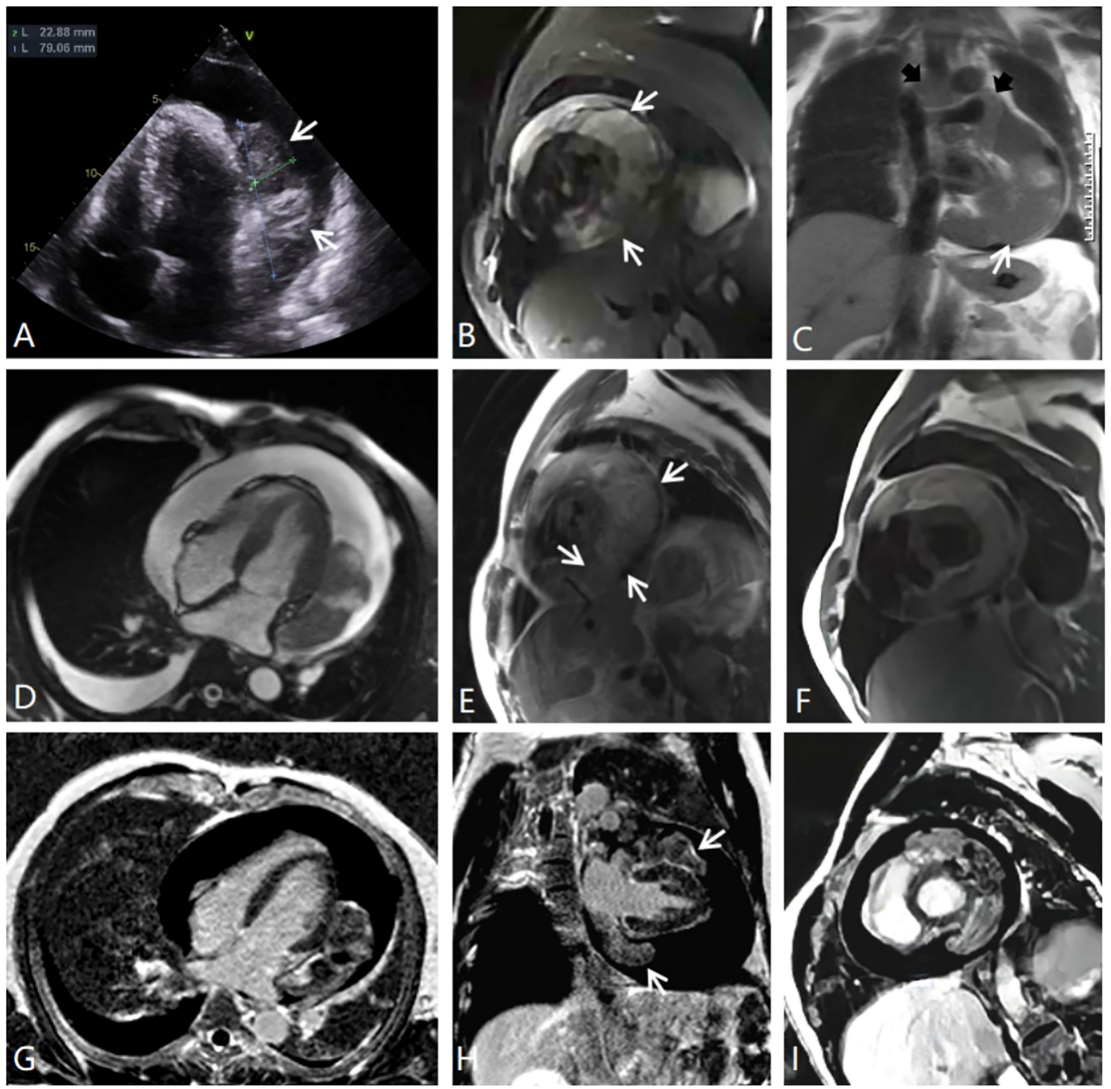
**(A)** Echocardiography. **(B)** MRI fat suppression image, short axial position of the left ventricle. **(C)** Half-Fourier single-shot turbo spin-echo MRI coronal image showing the tumor-encased superior vena cava, ascending aorta, and pulmonary trunk *(black arrows)*. **(D)** MRI cine sequence, four-chamber position. **(E)** MRI T2-weighted image, short axial position. **(F)** MRI T1-weighted image, short axial position. **(G–I)** MRI delayed enhancement, four-chamber position, two-chamber position, and short axial position. There is an inhomogeneous, enhancing intrapericardial mass surrounded by fluid, with no intracavitary or endoluminal invasion.

We aim to follow the CARE guidelines for cases reports. A 34-year-old man was admitted to the hospital with chest tightness and a cough that had been present for 6 days. He described having dyspnea that was gradually worsening. His other complaints included bloating, acid reflux, and heartburn. His blood pressure was 139/97 mm Hg, his heart rate was 85 beats/min, and his oxygen saturation was 98% with supplemental oxygen. His Holter monitoring showed sporadic atrial premature beats, sustained ST-T changes, and an abnormal Q wave of the posterior wall. Laboratory investigations demonstrated an elevated white blood cell count of 13.90 × 10^9^/L, a D-dimer level of 5100 ng/mL, and an N-telencephalon sodium peptide precursor (B-type natriuretic peptide) level of 843.20 pg/mL; however, the level of troponin-T was normal. The patient’s medical history and physical examination showed no significant abnormalities. An echocardiogram revealed a substantial amount of pericardial effusion and a 7.9 × 2.3 cm^2^ heterogeneous hypoechoic mass located in the left visceral pericardium. The mass was found to have a broad base and to be adjacent to the left ventricle and atrium (**Figure 1A**).

**Figure 2:**
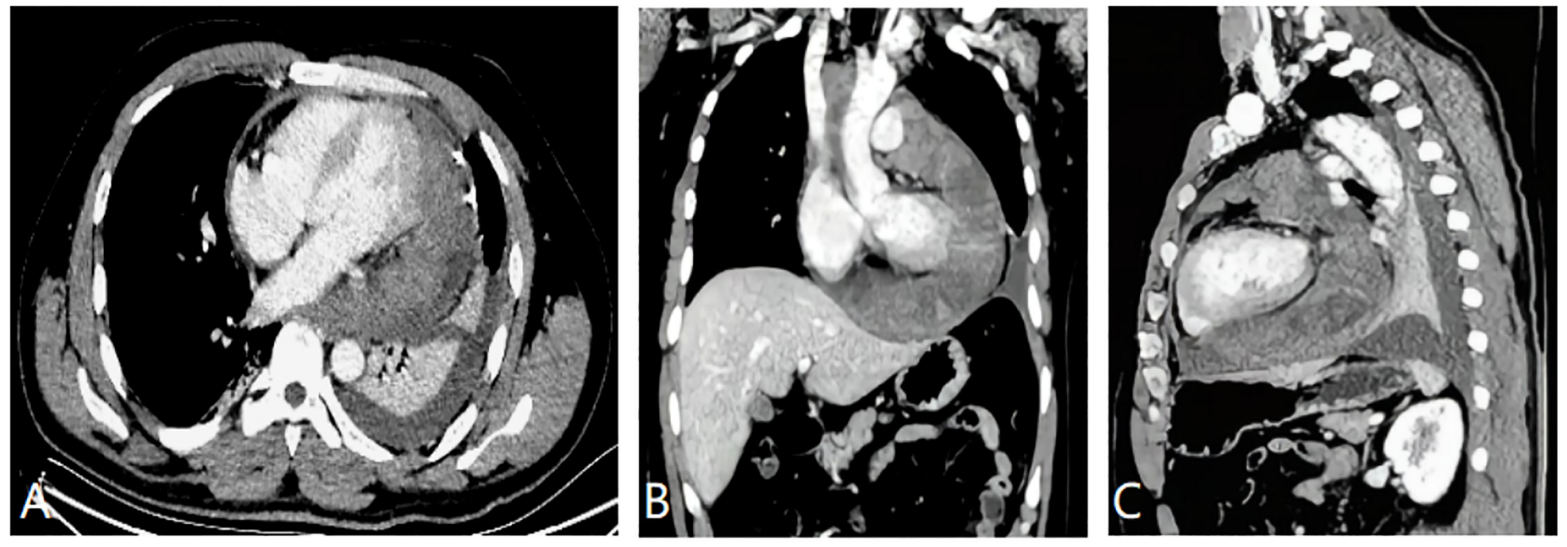
**(A)** Mildly enhanced axial CT image showing the large heterogenous left pericardial mass. There was a slight pericardial effusion. **(B)** Enhanced CT image of the coronal reconstruction showing the tumor circumscribing the ascending aorta, the superior vena cava, and the pulmonary artery. **(C)** CT image of the sagittal reconstruction showing the mass invading the pericardium, which presented uneven thickening andnodular changes.

CMRI and CT examinations were requested for the further diagnosis and assessment of the relationship between the tumor and its adjacent structures. Both of these imaging studies noted a large mass situated superior and inferior to the left side of the heart and adhering to the left ventricular free wall. The tumor completely covered the left atrium, superiorly extended between the ascending aorta and the superior vena cava, and partly encased the pulmonary artery trunk and the left pulmonary artery. In addition, CMRI showed that the irregular soft-tissue mass was asymmetrically hypointense on T1-weighted images and heterogeneously hyperintense on T2-weighted images and fat suppression images (**Figures 1B–F**). The first-pass perfusion images showed that there was no enhancement during the early phase; however, during the balance phase, the lesion appeared slight enhancement. Delayed enhancement images showed that part of the tumor presented mild to moderate patchy enhancement (**Figures 1G–I**). There were large pericardial effusions and bilateral pleural effusions, primarily on the right side. The CT imaging showed that the pericardium was invaded by a cystic solid lesion, and it presented local, unevenly thickened, and nodular changes. After the injection of contrast medium, the solid composition and septa of the tumor were slightly enhanced (**Figure 2**). CT scans of the head, chest, abdomen, and pelvis were negative, with no evidence of metastasis. Pericardial drainage under ultrasound guidance was performed before CT examination to relieve the patient’s chest tightness symptoms, and examination of the bloody pericardial fluid found a few short spindle-shaped cells with dyskaryosis.

**Figure 3:**
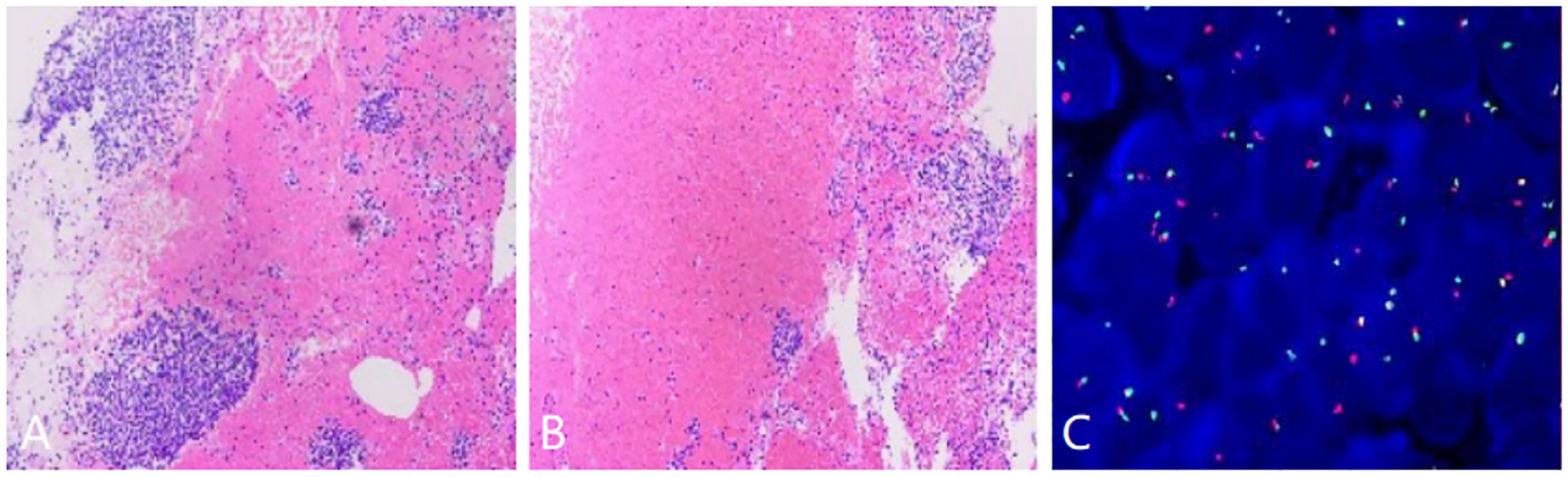
**(A, B)** Histopathological examination of the tumor tissue samples showing the fascicles of the spindle cells, **(C)** Fluorescence in situ hybridization demonstrating a separated red and green signal pattern indicative of an SS18 (SYT) gene rearrangement.

The patient subsequently underwent a CT guided percutaneous needle puncture biopsy in the CT room. Histopathological examination of the tissue samples showed spindle cell tumors with a typical structure of hematoxylin-eosin staining arranged in vague fascicles or sheets, which was most compatible with a diagnosis of monophasic SS (**Figures 3A, B**). Immune and molecular pathology results were as follows: AE1/AE3(CK) (individual +), EMA (+), Nestin (+), SMA (weak +), CD 34 (-); CD56(56C04)*(+), SYN (-), INI-1*(+), and CD99 (+). Fluorescence *in situ* hybridization (FISH) testing was positive as an SS18 (SYT) break-apart probe found that the SS18 gene was broken, thereby further supporting the diagnosis of PSS (**Figure 3C**).

**Figure 4:**
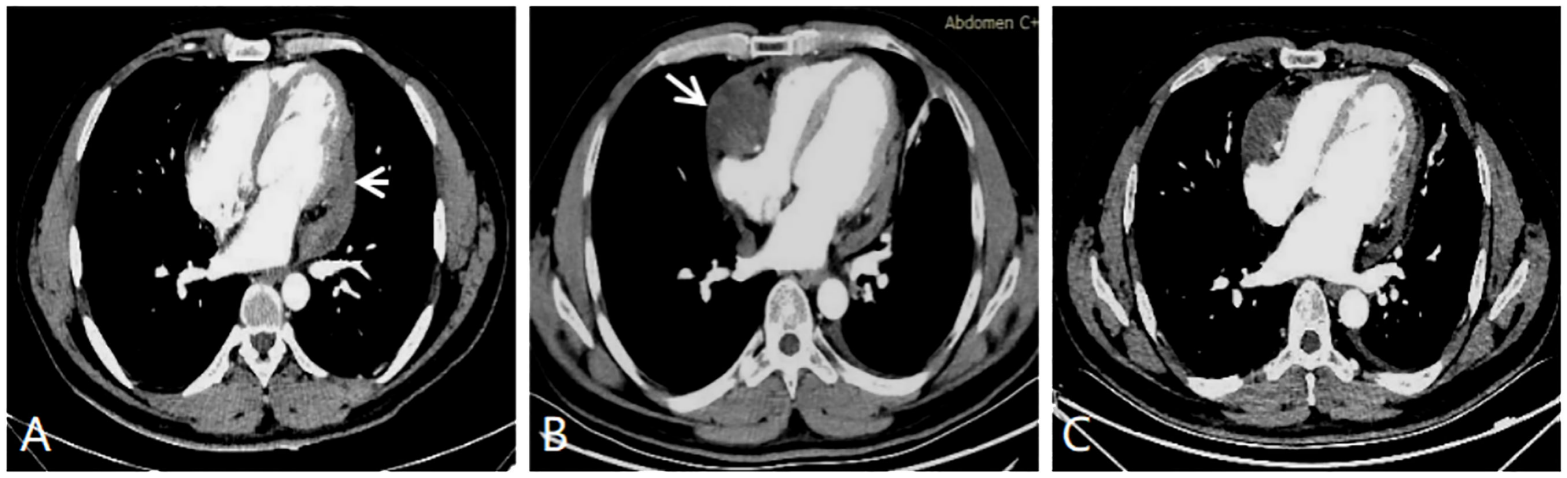
**(A)** CT enhanced image after six cycles of chemotherapy showing that the tumor has significantly regressed *(white short arrow).* **(B)** CT enhanced image tumor recrudescence in the contralateral pericardial cavity *(white long arrow)*. **(C)** CT enhanced image showing a slight reduction in the size of the primary and secondary tumors. This image was acquired after the recurrent tumor was treated with eight cycles of chemotherapy.

Given that the tumor has invaded the major vascular structures and could not be completely excised, the patient was not considered for surgical treatment. He elected to receive a doxorubicin-ifosfamide chemotherapy regimen in the oncology department. After six cycles of chemotherapy, the tumor had significantly shrunk, which was very well observed via CT imaging (**Figure 4A**). The patient then received one radiation treatment and was discharged home. Four months later, the patient came to the hospital for follow-up examination. CT images showed tumor relapse in the contralateral pericardial cavity. The tumor was located adjacent to the right atrium and the right ventricular junction, but there were no signs of recurrence at the site where the primary tumor had been located (**Figure 4B**). The patient underwent eight cycles of gemcitabine-based combination chemotherapy with acceptable tolerance. There was a slight reduction in tumor size (**Figure 4C**), but the second round of chemotherapy did not seem to be as effective as the first, possibly due to the change in the chemotherapy regimen. Unfortunately, the patient died 17 months after receiving his diagnosis and initiating chemotherapy.

## Discussion

SS is an aggressive neoplasm that originates from the primitive mesenchymal cells with epithelial differentiation potential, and it differentiates into neoplastic cells that are similar to synovial tissue. The etiology and pathogenesis of SS remains unknown, but it is characterized by a t(X;18)(p11.2;q11.2) chromosomal translocation, and about 95% of SS cases involve a t(X;18) translocation (4). The diagnosis of SS is mainly based on histopathology, immunophenotype, and specific SS18 (SYT)-SSX fusion gene changes. SS is generally composed of spindle cells, epithelial cells, or both, which are identified under a microscope. The diffusely growing tumor cells are arranged in sheets or bundles with or without cellular atypia, with scant cytoplasm and many mitotic figures. SS is classified into subtypes in accordance with the proportion, distribution, and differentiation of epithelial cells and spindle cells; these subtypes include the biphasic type, the monophasic epithelial type, the monophasic fiber type, and the low differentiation type. The biphasic type is more common with PSS, approaching a level of almost 50% of cases (5). Immunohistochemistry is commonly performed to detect the expression of cytokeratin, epithelial cell membrane antigen, and vimentin, which are sensitive biologic markers for the diagnosis of SS. At present, the SS18-SSX fusion gene has not been identified in tumors other than SS (6), so FISH gene detection has become the specific molecular diagnostic indicator of SS.

SS is typically located in the extremities, but it is occasionally found in the head and neck, the eye sockets, the lungs, the chest wall, and the mediastinum. Primary PSS is extremely rare, with only in a few dozen cases reported in the literature (7). PSS has a male preponderance; the median age of diagnosis is 32 years, and the mean tumor size is 10.6 cm (8). Patient symptoms are often nonspecific, and some patients appear to have no symptoms, with the diagnosis being made incidentally or not until the disease is at an advanced stage. The most common symptoms are dyspnea, chest pain, cough, palpitations, fever, fatigue, and other similar issues. Our patient was 34 years old, which corresponds with the median age as reported in the literature. He presented with dyspnea followed by chest tightness and cough, and these symptoms may be caused by tumor and pericardial effusion compression.

PSS on echocardiography usually presents as an irregular, lobulated, inhomogeneous hypoechoic mass; and the tumor is mostly accompanied by pericardial effusion and thickening, usually with a broad base, and only approximately 24.1% of tumors are pedicled (9). A previous report suggested that the diagnostic value of echocardiography may be limited when the pericardial tumor presents simultaneously with a massive pericardial effusion (2). The tumor in our case exhibited these echocardiography characteristics, but it was accurately detected by transthoracic echocardiography; this may be because the tumor had more solid components, thus resulting in a marked echocardiographic contrast. Although the ultrasound images of PSS are nonspecific and of limited diagnostic value, ultrasound is still often used as a first-choice radiologic modality for tumor screening and late sequential evaluation. As an imaging technique, it can depict thickened pericardium, pericardial effusion, and cardiac morphology, movement and function.

CT scanning can provide detailed information about the features of PSS and its relationship with surrounding structures; assess the involvement of the mediastinum, lung, and pleura; and identify lymph nodes and distant metastases. PSS is seen as an equal or slightly higher-density mass with clear or unclear boundaries on CT images. In some cases, wall nodules, necrosis, cystic degeneration, or hemorrhage may be seen in the lesion. The literature has reported marginal calcification being found in SS, which is helpful for the differential diagnosis (10). Only with plain CT scanning, it is difficult to distinguish the tumor body from normal myocardial tissue. If there is a large amount of pericardial effusion, it is easy to miss the diagnosis of PSS.

CMRI is a superior noninvasive diagnostic tool: it provides higher resolution for better characterization of details in patients with PSS, and it can detect invasion into the myocardium and other adjacent structures. CMRI can also accurately provide additional information related to cardiac and pericardium about structure or function. PSS on T1-weighted MRI often shows tumor parenchyma as an uneven equal signal, and T2-weighted images show mixed signals dominated by high signals. In other words, multiple T2 signals can appear within the tumor. This may involve “triple signals,” such as a low signal (calcification), a slightly higher signal (the tumor’s solid component), and an obviously high signal (liquefactive necrosis or cystic degeneration) (11). The solid component and the fibrous septa of the tumor are usually mildly enhanced, but the cystic necrotic areas are not enhanced. The cystic necrosis component may be associated with the rapid growth of the tumor.

In the present case, CT showed an irregular, cystic, solid mass with unclear boundaries and uneven density. However, no marginal calcification was found within the tumor, which differs from what has been previously reported in the literature (10). CMRI demonstrated mixed signals including slightly hyper-T2 signal intensity and obvious hyper-T2 signal intensity, which revealed different components within the tumor. This patient’s tumor also contained mildly enhanced solid components and septa and unenhanced cystic areas. CT and CMRI showed that the mass had extended into the mediastinum in a manner similar to the drilling of holes, which is a characteristic growth pattern of PSS as the literature has reported (12).

The differential diagnosis of PSS includes metastasis, pericardial mesothelioma, pericardial teratoma, and other subtypes of pericardial sarcomas (e.g., angiosarcoma, liposarcoma, rhabdomyosarcoma, undifferentiated sarcoma). The most common cardiac metastases are lung and breast cancer, however, known history of primary neoplasms is the main basis for differential diagnosis. Both primary and metastatic tumors can lead to bloody pericardial effusion. Metastatic pericardial effusion usually develops rapidly, prone to massive pericardial effusion, even causing pericardial tamponade, and easy to relapse after drainage.

Patients with PSS have extremely poor prognoses and are prone to relapse. Surgical resection remains the preferred treatment, although it is difficult and carries significant risks. A statistical study showed that the 2-year survival rates of complete and incomplete resection were 75.2% and 55.0% (13). The surgical resection of PSS can significantly improve patients’ survival, especially when the resection of the tumor is complete. Both postoperative chemoradiotherapy and chemotherapy alone are effective; they encourage tumor regression and prolong patients’ lives. Alternatively, radiotherapy alone hardly seems to improve the patient’s prognosis, and some cardiac complications related to such treatment need to be considered (14, 15). Cardiac transplantation is also an option when extensive infiltration of the tumor is involved. However, a study reported that there was no significant improvement in the survival rate of patients with primary cardiac sarcoma after heart transplantation as compared with traditional treatment (16). Our patient was not considered for heart transplantation because of the lack of donors. After six cycles of chemotherapy alone, our patient’s tumor was significantly reduced, which demonstrated that chemotherapy alone can be effective. Although the tumor did recur later, the patient survived for 17 months, which is significantly longer than the mean survival length of 6 months reported in the literature (17).

## Conclusion

PSS is a rare clinical disease, but the symptoms and presentation are usually cryptic and atypical, such as the case of this study only presented dyspnea and cough. Accordingly, the diagnosis and management of PSS are complex and need multidisciplinary collaboration and multimodal imaging examinations. It is necessary to continuously report and accumulate information about new PSS cases to develop a more comprehensive understanding of this disease’s clinical characteristics, pathological diagnosis, and multimodal imaging features. Multimodality cardiac imaging includes echocardiography, CT, and CMRI, all of which are critical for the diagnosis, management, staging, and prognosis of PSS. In this article, we discuss the role of each imaging modality and the features of PSS in the hope of providing valuable reference information for affected patients in the future.

## Data availability statement

The original contributions presented in the study are included in the article/supplementary material. Further inquiries can be directed to the corresponding author.

## Ethics statement

Written informed consent was obtained from the necessary individual(s) for the publication of any potentially identifiable images or data included in this article.

## Author contributions

HJ and WZ analyzed the data and image acquisition, and KW designed the study. All authors revised the manuscript. They all commented and approved the final version of the manuscript. They have contributed equally to this work.
